# Optimized Synthesis and Properties of Dumbbell-Shaped NaYF_4_:Yb,Tm@NaGdF_4_:Eu@NH_2_-MIL-53(Fe) Core–Shell Nanocomposite

**DOI:** 10.3390/molecules31193412

**Published:** 2026-09-24

**Authors:** Xinyao Su, Wen Guo, Qunming Zhou, Tingting Xie, Lingyan Wu, Shangyang Wang, You Fu, Shurong Li, Wei Peng, Yun Qi, Lingyan Zhang, Peijun Meng

**Affiliations:** 1 School of Public Health, Baotou Medical College, Inner Mongolia University of Science & Technology, Baotou 014040, China; 2024270147@stu.btmc.edu.cn (X.S.); gmaomao0029@163.com (W.G.); 18470412753@163.com (Q.Z.); x19847202910@163.com (T.X.); 18534637270@163.com (L.W.); 18784021878@163.com (S.W.); 13734943764@163.com (Y.F.); 102009006@btmc.edu.cn (S.L.); 102024014@btmc.edu.cn (W.P.); qiyun@btmc.edu.cn (Y.Q.); 2 Public Health Risk Prevention and Control Integrated Scientific and Technological Innovation Platform Based on “One Health” of Inner Mongolia Autonomous Region, Baotou 014040, China

**Keywords:** upconversion nanoparticles, metal–organic framework, nucleocapsid structure, multifunctional nanomaterials

## Abstract

Cancer theranostics urgently demands multifunctional nanomaterials capable of simultaneously enabling high-sensitivity imaging and multimodal synergistic therapy. In this study, a three-layer core–shell nanocomposite, NaYF_4_:Yb,Tm@NaGdF_4_:Eu@NH_2_-MIL-53(Fe), was designed and synthesized. The NaYF_4_:Yb,Tm nanoparticle core provides upconversion luminescence upon near-infrared (NIR) excitation and can serve as an excitation source for photodynamic therapy. An epitaxially grown NaGdF_4_:Eu intermediate shell was introduced to enhance luminescence and provide T_1_-weighted magnetic resonance imaging (MRI) capability. The outermost NH_2_-MIL-53(Fe) metal–organic framework shell was subsequently coated to provide potential chemodynamic therapy (CDT) capability. The effects of the Eu^3+^ doping concentration, reaction temperature, reaction time, and oleic acid (OA)/1-octadecene (ODE) ratio on the anisotropic growth into a dumbbell-like morphology were examined to determine the optimal conditions. Under the optimal conditions (10 mol% Eu^3+^, 300 °C, 60 min, OA/ODE = 4:16), core–shell nanocrystals with regular morphology, uniform size, and the strongest upconversion luminescence were obtained. Polyvinylpyrrolidone (PVP)-mediated surface functionalization successfully induced the in situ growth of the NH_2_-MIL-53(Fe) outer shell, thereby constructing a three-layer core–shell nanocomposite as a potential theranostic platform. Various characterizations confirmed that the resulting composite integrates upconversion luminescence, paramagnetism, and hydroxyl radical generation capability, suggesting potential for NIR-driven dual-modal imaging-guided synergistic therapy.

## 1. Introduction

Cancer remains one of the leading causes of death worldwide. According to 2022 statistics from the World Health Organization, approximately 20 million new cancer cases and nearly 10 million cancer-related deaths occur globally each year [1]. Conventional chemotherapy is often limited by drug resistance, toxic side effects, and tumor heterogeneity, which falls far short of the urgent demand for precision medicine for personalized, highly effective, and low-toxicity therapeutic strategies [2,3]. Cancer theranostics, which integrates diagnostic and therapeutic functions into a single platform, is expected to effectively address these challenges by enabling real-time monitoring of therapeutic responses and personalized treatment adjustments [4]. With the rapid development of nanotechnology, theranostic nanoplatforms that integrate drug delivery and imaging functions can achieve sensitive early-stage diagnosis, maximize drug accumulation at lesion sites, and trigger precise treatment of diseased tissues through external or internal stimuli, thereby enhancing therapeutic efficacy and reducing systemic toxic side effects. This fully demonstrates the enormous potential of nanomaterials in advancing personalized precision medicine for cancer [5,6]. Because a single nanoparticle can hardly satisfy multiple functions simultaneously, nanocomposites composed of two or more functional building blocks have attracted increasing attention [7]. Among them, the combination of upconversion nanoparticles (UCNPs) with metal–organic frameworks (MOFs) is attractive owing to the potential complementarity between their imaging and therapeutic functions [8].

UCNPs can convert near-infrared (NIR) light into visible or ultraviolet emission and possess narrow emission bands, long luminescence lifetimes, high photostability, and low autofluorescence background [9,10,11]. However, their large surface-to-volume ratio exposes many lanthanide ions to surface defects, organic ligands, and solvent molecules, leading to severe fluorescence quenching and reduced luminescence efficiency [12]. Core–shell engineering can effectively suppress surface quenching and regulate dopant distribution [13,14]. In particular, incorporating Gd^3+^ into the shell can shorten the T_1_ relaxation time through its paramagnetism, providing potential magnetic resonance imaging (MRI) capability [15]. Eu^3+^ can also be introduced into the NaGdF_4_ shell as a dopant to modulate the host lattice. Owing to these features, UCNPs have shown potential in biomarker detection, photodynamic therapy (PDT), drug delivery, and multimodal imaging [16,17,18,19].

MOFs are porous crystalline materials assembled from metal ions or cluster nodes and organic bridging ligands [20,21]. They have attracted interest in biomedicine because of their high porosity, large specific surface area, good biocompatibility, and tunable drug-loading and release properties [22,23]. MIL-53(Fe), an iron-based MOF, has been reported to possess a high specific surface area and to degrade in the acidic tumor microenvironment, releasing Fe^3+^ for potential chemodynamic therapy (CDT) [24]. However, MIL-53(Fe) alone lacks imaging capability. Therefore, combining UCNPs with MIL-53(Fe) may create functional complementarity: UCNPs provide upconversion luminescence imaging and NIR-triggered PDT potential, while MIL-53(Fe) offers drug loading, pH-responsive release, and CDT potential. Recent studies have explored UCNP/MIL-53(Fe) systems for tumor theranostics [25,26]. Although promising, further optimization is still needed in terms of MRI sensitivity, multi-color fluorescence tracking, and multifunctional synergism.

Based on the above design concepts, this study designed and synthesized a three-layer core–shell nanocomposite, NaYF_4_:Yb,Tm@NaGdF_4_:Eu@NH_2_-MIL-53(Fe), as a material platform for potential NIR-light-mediated imaging and therapy. In this composite, the NaYF_4_:Yb,Tm core generates upconversion luminescence under 980 nm NIR excitation; the NaGdF_4_:Eu intermediate layer introduces Gd^3+^ for potential T_1_-weighted MRI and Eu^3+^ as a dopant to modulate the host lattice; and the NH_2_-MIL-53(Fe) outer shell provides a high specific surface area and Fe^3+^ sites for potential CDT via Fenton-like reactions. In the experiments, NaYF_4_:Yb,Tm@NaGdF_4_:Eu core–shell upconversion nanoparticles were first prepared by a solvothermal method. The shell reaction parameters, including the Eu^3+^ doping amount, reaction temperature, reaction time, and OA/ODE volume ratio, were optimized to obtain well-defined morphology and enhanced upconversion luminescence; this optimization serves as a synthetic basis for constructing the final composite. Subsequently, PVP was modified onto the surface to improve aqueous dispersibility and introduce coordination sites, thereby inducing in situ coating of the NH_2_-MIL-53(Fe) shell to form the three-layer core–shell nanocomposite. The morphology, structure, and upconversion luminescence properties were characterized by X-ray diffraction (XRD), transmission electron microscopy (TEM), and fluorescence spectroscopy. This work focuses on synthesis and fundamental characterization; biological evaluations such as cytotoxicity, drug loading, CDT/PDT, and MRI will be addressed in future studies.

## 2. Results

### 2.1. Synthetic Strategy

The synthetic route of the dumbbell-shaped core–shell NaYF_4_:Yb,Tm@NaGdF_4_:Eu@N H_2_-MIL-53(Fe) nanoparticles is illustrated in Figure 1. First, oleic acid-capped NaYF_4_:Yb,T m cores were prepared, and a NaGdF_4_:Eu shell was epitaxially grown onto them to yield oleate-coordinated core–shell upconversion nanoparticles (NaYF_4_:Yb,Tm@NaGdF_4_:Eu). Subsequently, the oleic acid ligands were removed by protonating the carboxyl groups with dilute HCl, producing hydrophilic bare nanoparticles. These bare nanoparticles were then surface-modified with polyvinylpyrrolidone (PVP), which enriched Fe^3+^ ions in the system through the strong coordination of the PVP pyrrolidone groups. Afterward, the organic ligand 2-aminoterephthalic acid (NH_2_-H_2_BDC) was introduced to induce the heterogeneous nucleation and growth of NH_2_-MIL-53(Fe) on the nanoparticle surface. Using the upconversion nanoparticles as growth substrates effectively suppressed the homogeneous nucleation of the MOF in solution, ultimately affording the desired hierarchical core–shell nanocomposite NaYF_4_:Yb,Tm@NaGdF_4_:Eu@NH_2_-MIL-53(Fe).

### 2.2. Morphology and Structural Analysis of NaYF_4_:Yb,Tm

Figure 2 presents the transmission electron microscopy (TEM) and X-ray diffraction (XRD) characterization results of the NaYF_4_:Yb,Tm synthesized in the first step of this study. The results reveal that the as-prepared NaYF_4_:Yb,Tm upconversion nanomaterials possess a hexagonal phase with uniform morphology and good dispersibility and exhibit an average particle size of 26.50 ± 0.98 nm. The XRD diffraction peak positions of NaYF_4_:Yb,Tm are in good agreement with those of the standard hexagonal-phase NaYF_4_ (JCPDS No. 28-1192), indicating the successful synthesis of the hexagonal-phase NaYF_4_:Yb,Tm and laying a favorable foundation for the subsequent NaGdF_4_:Eu shell coating experiment.

### 2.3. Influence of Eu^3+^ Doping Amount on the Morphology and Properties of NaYF_4_:Yb,Tm@NaGdF_4_:Eu

TEM images of the NaYF_4_:Yb,Tm@NaGdF_4_:Eu nanomaterials synthesized with different Eu^3+^ doping amounts are presented in Figure 3A. All samples exhibited a distinct core–shell structure, in which the NaGdF_4_:Eu shell was epitaxially coated onto the surface of the NaYF_4_:Yb,Tm core, leading to a significant increase in particle size compared with the bare core. Nevertheless, the morphology varied with the doping amount. At low doping levels (5% and 10%), the particles displayed regular morphology and good monodispersity. When the Eu^3+^ doping amount was increased to 15% and 20%, the particles underwent anisotropic growth and adopted a dumbbell-like morphology, accompanied by a decline in coating uniformity and monodispersity. This anisotropic growth originated from the lattice mismatch between the NaGdF_4_:Eu shell and the NaYF_4_:Yb,Tm core, as well as the differences in ionic radii among Y^3+^, Gd^3+^, and Eu^3+^, indicating that the lattice strain at the core–shell interface induced by high-concentration Eu^3+^ doping compromised the uniformity of the epitaxial shell growth.

The XRD characterization in Figure 3B further revealed the crystal phase structure. The diffraction peaks of the NaYF_4_:Yb,Tm core matched perfectly with those of hexagonal-phase β-NaYF_4_ (JCPDS No. 28-1192) without any impurity peaks, indicating a pure hexagonal phase with high crystallinity that provided stable seeds for the epitaxial shell growth. After coating with the NaGdF_4_:Eu shell, the main diffraction peaks were in good agreement with those of β-NaGdF_4_ (JCPDS No. 27-0699), confirming that Eu^3+^ ions were successfully incorporated into the NaGdF_4_ lattice without introducing any impurity phase. As the Eu^3+^ doping amount increased from 5% to 20%, the diffraction peak intensities gradually decreased, and the peaks became slightly broadened. This was attributed to the lattice distortion arising from the slight difference in ionic radii between Eu^3+^ (0.950 Å) and Gd^3+^ (0.938 Å), which led to a slight reduction in crystallinity. To quantify this effect, the crystallite sizes of the samples with different Eu^3+^ concentrations were calculated from the (100) diffraction peak using the Scherrer equation:

$$D=\frac{K\lambda }{\beta \cos\theta }$$

where *D* is the crystallite size, *K* = 0.89, *λ* = 1.54056 Å is the wavelength of the incident X-rays, *β* is the full width at half maximum (FWHM) in radians, and *θ* is the Bragg angle (peak position in the diffractogram). With increasing Eu^3+^ concentration from 5% to 20%, the crystallite size decreased from 22.13 nm (5%) to 22.06 nm (10%), 16.80 nm (15%), and 8.46 nm (20%). At low Eu^3+^ concentrations (5% and 10%), the crystallite size remained almost unchanged, whereas it decreased significantly at higher concentrations (15% and 20%), suggesting that a high Eu^3+^ content induces more pronounced lattice distortion and inhibits crystallite growth. In contrast, the overall particle morphology observed by TEM remained largely unchanged because the morphology is mainly governed by the reaction kinetics and shell growth process, whereas XRD reflects the size of crystalline domains. This observation was consistent with the anisotropic growth trend revealed by TEM.

The upconversion luminescence spectra of NaYF_4_:Yb,Tm NaGdF_4_:Eu synthesized with different Eu^3+^ doping amounts, recorded under 980 nm excitation, are shown in Figure 3C. All samples exhibited characteristic emission peaks of Tm^3+^ centered at 348, 364, 454, and 478 nm, corresponding to the ^3^P_0_→^3^F_4_, ^1^D_2_→^3^H_6_, ^1^D_2_→^3^F_4_, and ^1^G_4_→^3^H_6_ transitions, respectively. Compared with the bare core, the emission intensities of all peaks were substantially enhanced after coating with the NaGdF_4_:Eu shell, which can be attributed to the effective passivation of surface defects on the core by the shell layer, thereby suppressing the energy loss caused by surface quenching. As the Eu^3+^ doping concentration increased from 5 mol% to 20 mol%, the upconversion luminescence intensity first increased and then decreased, reaching a maximum at 10 mol%. This optimal concentration reflects a balance between morphology and energy transfer: excessive Eu^3+^ doping leads to the accumulation of lattice strain, which deteriorates the particle morphology and increases defects at the core–shell interface, thereby aggravating the non-radiative recombination of Tm^3+^ and weakening the luminescence.

### 2.4. Influence of OA/ODE Volume Ratio on the Morphology and Properties of NaYF_4_:Yb,Tm@NaGdF_4_:Eu

For the case with the shell’s Eu^3+^ doping amount fixed at 5%, the TEM images of NaYF_4_:Yb,Tm@NaGdF_4_:Eu nanoparticles synthesized at different OA/ODE volume ratios are shown in Figure 4A. At OA/ODE = 8:12, the core–shell nanocrystals exhibited incomplete coating and heterogeneous morphology, along with a relatively low particle density. A large number of free ellipsoidal NaGdF_4_:Eu particles generated through homogeneous nucleation were also present, leading to a highly non-uniform particle size distribution. This phenomenon can be attributed to the strong adsorption of the high-concentration OA onto the crystal surfaces, which excessively passivated the crystal facets. This not only significantly suppressed the heterogeneous epitaxial growth of the shell precursors on the core surface but also promoted the homogeneous nucleation of the precursors. The free particles grew into an ellipsoidal shape under the selective adsorption of OA, resulting in the coexistence of core–shell particles and free particles. When the ratio decreased to 6:14, the morphology and dispersibility improved markedly, with the particles exhibiting a well-defined hexagonal-phase profile and almost no residual free particles. However, the edges of some particles remained slightly blurred, and the size uniformity had not yet reached the optimal level. At OA/ODE = 4:16, the nanocrystals displayed a highly regular hexagonal-phase structure with a narrow size distribution, excellent monodispersity, and negligible agglomeration, representing the best morphology and dispersibility among this series. At this ratio, the OA concentration achieved a balance between passivation and growth. Moreover, because excess NaOH was introduced during the synthesis to deprotonate oleic acid (OAH + OH^−^ → OA^−^ + H_2_O), a large amount of the generated OA^−^ preferentially and firmly passivated the (100) side facets, keeping the (001) facets relatively active and thereby promoting the uniform longitudinal growth of the shell along the c-axis. When the ratio was further reduced to 2:18, the monodispersity decreased significantly, with pronounced agglomeration, a broadened size distribution, and irregular hexagonal outlines or even slight morphological distortion observed in some particles. This deterioration occurred because the OA concentration was too low to effectively passivate and protect the crystal surfaces. The insufficient ligand coverage led to a decline in colloidal stability, making the particles prone to agglomeration and fusion, and consequently worsening the morphology and dispersibility.

XRD patterns (Figure 4B) of the NaYF_4_:Yb,Tm@NaGdF_4_:Eu core–shell nanocrystals synthesized at different OA/ODE volume ratios all matched the standard hexagonal-phase NaGdF_4_ (JCPDS No. 27-0699), without any impurity peaks. As the OA/ODE ratio decreased from 8:12 to 4:16, the diffraction peaks gradually sharpened, indicating that a moderate reduction in OA concentration facilitated ordered crystal growth and significantly enhanced crystallinity. Upon further decreasing the ratio to 2:18, the diffraction peaks became slightly broadened, and their intensities decreased slightly, which was attributed to insufficient passivation of crystal facets, increased defect density, and particle aggregation caused by colloidal instability at an excessively low OA concentration. Moreover, the intensity ratio of the (101) to (110) diffraction peaks varied systematically with the decrease in OA/ODE ratio, directly reflecting the differential regulation of OA concentration on the relative growth rates of different crystal facets.

The fluorescence spectra (Figure 4C) further revealed that the upconversion luminescence intensity exhibited a trend of first increasing and then decreasing with the variation in OA/ODE ratio, reaching a maximum at OA/ODE = 4:16. Under this condition, the intact core–shell structure and high crystallinity effectively reduced surface defects and non-radiative relaxation, thereby significantly enhancing the luminescence efficiency. In contrast, at excessively high or low OA ratios, non-radiative transitions were aggravated due to inhomogeneous coating with coexisting free particles or particle agglomeration, respectively, leading to a marked decrease in luminescence intensity.

Taken together, OA/ODE = 4:16 was identified as the optimal ratio for this system, yielding core–shell nanocrystals with regular morphology, high crystallinity, and excellent luminescence properties, thus laying a favorable foundation for subsequent applications.

### 2.5. Influence of Reaction Temperature on the Morphology and Properties of NaYF_4_:Yb,Tm@NaGdF_4_:Eu

Considering the case with the Eu^3+^ doping amount fixed at 10% and the OA/ODE ratio at 4:16, TEM images of NaYF_4_:Yb,Tm@NaGdF_4_:Eu nanoparticles synthesized at different reaction temperatures are presented in Figure 5A. At 280 °C, only a large number of fine free particles were observed, and no complete shell coating was formed, indicating that this temperature was insufficient to provide the activation energy required for the epitaxial growth of shell precursors onto the core surface. The precursors tended to nucleate directly in the solution rather than undergo oriented deposition on the seed surface. Moreover, the slow release of fluoride ions at the relatively low temperature could not supply adequate ionic sources for sustained epitaxial shell growth, further restricting the formation of core–shell structures. As the temperature was raised to 290 °C, the shell growth mode transitioned from homogeneous nucleation to heterogeneous epitaxy. A distinct coating appeared on most core surfaces, and a core–shell structure preliminarily formed, accompanied by a significant increase in particle size. However, a certain amount of free α-NaGdF_4_:Eu particles remained in the system, and the morphology was heterogeneous, with some particles incompletely coated, reflecting the persistent competition between homogeneous nucleation and heterogeneous growth. When the temperature reached 300 °C, heterogeneous epitaxial growth became completely dominant, and homogeneous nucleation was effectively suppressed. The shell grew uniformly and epitaxially on the core surface, yielding a well-defined hexagonal-phase core–shell structure with regular morphology and good dispersibility. Upon further increasing the temperature to 310 °C, the nanocrystal morphology transformed from regular hexagonal prisms into a typical dumbbell shape, characterized by a constricted middle and thickened ends, exhibiting pronounced anisotropic growth. This occurred because the passivation of the (001) end facets by oleic acid became destabilized at the high temperature, significantly enhancing the reactivity of the end facets, whereas the oleate ions (OA^−^) remained stably adsorbed on the (100) side facets, maintaining their passivating effect. Consequently, preferential longitudinal growth of the shell along the c-axis was promoted. Meanwhile, the strongly binding OA^−^ could strip rare earth ions from the side facets at the elevated temperature, causing partial dissolution of the core side surfaces and further aggravating the morphological evolution.

The XRD patterns of NaYF_4_:Yb,Tm@NaGdF_4_:Eu synthesized at different reaction temperatures are presented in Figure 5B. At 280 °C, the pattern simultaneously exhibited characteristic diffraction peaks of both the core and the shell, but the peaks were broad and of relatively low intensity. Combined with TEM observations, this indicated that the shell predominantly underwent homogeneous nucleation at this temperature, forming mainly metastable cubic-phase or low-crystallinity NaGdF_4_:Eu particles, with limited epitaxial shell growth on the core. Upon increasing the temperature to 290 °C, the diffraction peaks intensified and became sharper, and their match with the standard hexagonal-phase NaGdF_4_ (JCPDS No. 27-0699) improved, indicating the preliminary formation of a core–shell structure. However, a small amount of incompletely crystallized precursor still remained, resulting in slightly broadened peaks. At 300 °C, the shell diffraction peaks reached the highest intensity with sharp profiles and perfectly matched the standard card, while the core diffraction peaks remained clear without any impurity peaks, confirming the formation of a pure hexagonal-phase core–shell structure with excellent crystallinity. Upon further heating to 310 °C, the diffraction peak intensities decreased, and the peaks broadened slightly. Combined with the dumbbell-like morphology observed in TEM, this was attributed to the abnormal growth of certain crystal facets caused by the desorption of surfactants at the high temperature. Meanwhile, the excessively high temperature also introduced a small number of lattice defects, leading to a slight decrease in crystallinity.

The upconversion fluorescence spectra of NaYF_4_:Yb,Tm@NaGdF_4_:Eu synthesized at different reaction temperatures are shown in Figure 5C. No obvious shifts were observed in the positions of any emission peaks, indicating that the reaction temperature did not alter the energy level structure of Tm^3+^ but primarily affected the luminescence intensity. As the temperature increased, the upconversion luminescence intensity exhibited a trend of first increasing and then decreasing. At 280 °C, the intensity was the lowest, which was attributed to incomplete shell coating, abundant surface and interfacial defects, and the dominance of non-radiative relaxation. At 290 °C, the intensity was significantly enhanced, indicating the preliminary formation of a core–shell structure, a reduction in defect density, and a certain degree of suppression of non-radiative transitions. At 300 °C, the intensity reached its maximum, with all characteristic emission peaks substantially enhanced and the highest upconversion luminescence efficiency achieved. At 310 °C, the intensity decreased, which originated from the excessively high temperature causing an increase in non-radiative recombination centers, thereby aggravating luminescence quenching.

In summary, the TEM, XRD, and fluorescence spectroscopy characterization consistently demonstrated that, with other conditions held constant, the NaYF_4_:Yb,Tm@NaGdF_4_:

Eu nanoparticles synthesized at a reaction temperature of 300 °C possessed regular morphology, uniform particle size, and the strongest upconversion luminescence intensity.

### 2.6. Influence of Reaction Time on the Morphology and Properties of NaYF_4_:Yb,Tm@NaGdF_4_:Eu

For the case with the Eu^3+^ doping amount fixed at 10%, the OA/ODE ratio at 4:16, and the reaction temperature at 300 °C, TEM images of NaYF_4_:Yb,Tm@NaGdF_4_:Eu nanoparticles synthesized at different reaction times are presented in Figure 6A. Figure 6(Aa) shows the TEM image of the nanocrystals obtained after 30 min of reaction. At this early stage of shell growth, the precursors did not achieve uniform epitaxial deposition on the core surface, resulting in incomplete coating. Meanwhile, a large number of free, nearly spherical α-NaGdF_4_:Eu nanocrystals existed in the system, and the core–shell nanomaterials displayed non-uniform size and morphology without the formation of a complete core–shell structure. Upon extending the reaction time to 60 min (Figure 6(Ab)), the shell achieved uniform epitaxial growth. The particles exhibited a well-defined hexagonal-phase structure with excellent monodispersity, very few free particles, and an average size of 36.55 ± 1.02 nm, marking the formation of a complete core–shell structure. A critical morphological transformation occurred at 90 min and beyond. As the reaction time further increased, the lattice mismatch strain energy induced by the ionic radius difference between the core (NaYF_4_:Yb,Tm) and the shell (NaGdF_4_:Eu) accumulated rapidly, compelling the system to release energy by adjusting the growth mode. NaYF_4_ crystals exhibit strong structural anisotropy: the side facets are {100} nonpolar planes with balanced surface charge and a chemically inert nature, whereas the top facets are {001} polar planes carrying net charge and possessing higher reactivity. This difference dictates the strain relaxation pathway—lateral growth along the nonpolar directions would continuously accumulate misfit strain at the rigid interface, raising the system energy; in contrast, longitudinal growth along the polar <001> direction can effectively release the strain energy through elastic relaxation along the c-axis. Consequently, the shell precursors preferentially undergo epitaxial deposition at the two ends (001 facets) of the nanocrystals to minimize the total energy of the system. Concurrently, the oleate ions (OA^−^) in the reaction system play a critical role in directing and amplifying this anisotropic growth: OA^−^ strongly and selectively binds to the {100} nonpolar side facets, forming a dense passivation layer under prolonged high-temperature conditions, which greatly suppresses lateral growth; in contrast, the {001} polar end facets remain weakly passivated and maintain high reactivity, continuously accepting new monomer deposition. Under the synergistic effect of these two mechanisms, the subsequently supplied NaGdF_4_:Eu monomers were almost exclusively deposited at the two ends, forming a dumbbell-shaped structure with enlarged ends and a constricted middle without etching. At this stage, the length of the core–shell nanoparticles increased to 49.36 ± 1.1 nm, while the average diameter at the middle was approximately 19.63 ± 1.0 nm and the width at the ends was approximately 30.19 ± 0.6 nm; the middle constriction originated from partial dissolution of the NaYF_4_:Yb,Tm core surface during shell growth. When the reaction was prolonged to 120 min, the anisotropic growth became more pronounced, accompanied by slight Ostwald ripening. The dumbbell morphology became even more distinct, but the monodispersity decreased to some extent.

The XRD patterns of the NaYF_4_:Yb,Tm@NaGdF_4_:Eu core–shell nanocrystals synthesized at different reaction times are shown in Figure 6B. All samples exhibited diffraction peaks consistent with those of β-NaGdF_4_ (JCPDS No. 27-0699), indicating that the reaction time did not alter the fundamental hexagonal-phase structure of the crystals. At a reaction time of 30 min, the diffraction peaks were relatively weak and broad, reflecting low crystallinity. When the reaction time was extended to 60 min, the diffraction peaks became significantly stronger and sharper, with no impurity peaks observed, and the crystallinity reached its optimum. Upon further prolonging the reaction time to 120 min, the diffraction peak intensities decreased, and the peaks broadened; meanwhile, impurity peaks that did not belong to the hexagonal main phase appeared in the pattern, indicating the formation of impurity phases. In summary, 60 min was determined to be the optimal reaction time for this system, yielding a core–shell structure with high crystallinity and a pure crystal phase.

The fluorescence spectra of NaYF_4_:Yb,Tm@NaGdF_4_:Eu synthesized at different reaction times are presented in Figure 6C. At a reaction time of 30 min, the fluorescence intensity showed a preliminary enhancement compared with the uncoated core sample. When the reaction time reached 60 min, the fluorescence intensity was substantially enhanced, indicating that the crystal growth tended toward completion under this condition and the surface defects were effectively passivated. As the reaction time was further extended to 90 and 120 min, the fluorescence intensity decreased, which could be attributed to increased defects or aggregation caused by excessive crystal growth.

In summary, through characterization by TEM, XRD, and fluorescence spectroscopy, it was determined that, with other conditions held constant, the NaYF_4_:Yb,Tm@NaGdF_4_:Eu nanoparticles synthesized at a reaction time of 60 min exhibited favorable morphology and uniform particle size.

The elemental composition and spatial distribution of the NaYF_4_:Yb,Tm@NaGdF_4_:Eu nanoparticles were characterized by scanning transmission electron microscopy–energy-dispersive X-ray spectroscopy (STEM-EDS) elemental mapping. As shown in Figure 7, the Gd signal was significantly enriched in the shell region, whereas the Eu signal was relatively weak. In the core region, signals of Y, Yb, and Tm were predominantly detected, with the Y signal being relatively strong, which was positively correlated with its higher doping concentration. The spatial distribution of these elements directly confirmed the designed core–shell heterostructure.

### 2.7. Synthesis and Properties of NaYF_4_:Yb,Tm@NaGdF_4_:Eu @NH_2_-MIL53(Fe)

To systematically investigate the removal of oleic acid (OA) ligands from the UCNP surface and the subsequent exchange with polyvinylpyrrolidone (PVP), the evolution of surface functional groups in OA, OA-UCNPs, OA-stripped UCNPs, pure PVP, and PVP-UCNPs was monitored by Fourier-transform infrared spectroscopy (FTIR). The corresponding results are presented in Figure 8E. In the spectrum of pure OA, the absorption peaks at 2921 cm^−1^ and 2852 cm^−1^ are assigned to the asymmetric and symmetric stretching vibrations of the methylene groups (-CH_2_-) of the long alkyl chain, respectively. The peak at 1707 cm^−1^ corresponds to the characteristic C=O stretching vibration of the free carboxyl group. In the spectrum of OA-UCNPs, the free carboxyl peak at 1707 cm^−1^ completely disappeared, while new peaks emerged at 1547 cm^−1^ and 1459 cm^−1^, attributed to the asymmetric and symmetric stretching vibrations of carboxylate (-COO^−^), respectively. This observation confirms that the OA molecules were coordinated to the UCNP surface in the form of carboxylate. After ligand stripping, the intensities of the methylene stretching vibrations and carboxylate stretching vibrations associated with OA were significantly weakened in the OA-stripped UCNPs. Pure PVP exhibited characteristic peaks at 1660 cm^−1^ and 1267 cm^−1^, which are assigned to the C=O stretching vibration of the pyrrolidone ring and the C–N stretching vibration, respectively. In the spectrum of PVP-UCNPs, the C=O and C-N stretching vibrations of PVP appeared at 1671 cm^−1^ and 1289 cm^−1^, respectively, exhibiting a blue shift relative to pure PVP. This blue shift is attributed to the disruption of intermolecular hydrogen bonds among PVP molecules and the interaction between the pyrrolidone ring and the UCNP surface. Meanwhile, the characteristic carboxylate peaks originating from OA largely disappeared. These results collectively confirm that the hydrophobic OA ligands were effectively removed from the UCNP surface, and hydrophilic PVP was successfully modified onto the surface. The PVP coating can adsorb Fe^3+^ ions and provide abundant active sites, thereby laying the foundation for the subsequent heterogeneous nucleation and growth of the NH_2_-MIL-53(Fe) shell onto the UCNP surface. Figure 8F displays the FTIR spectra of NH_2_-MIL-53(Fe) and UCNPs@NH_2_-MIL-53(Fe). In the spectrum of NH_2_-MIL-53(Fe), the broad absorption band centered at 3325 cm^−1^ is attributed to the N-H stretching vibration of the amino group (-NH_2_); the peaks at 1575 cm^−1^ and 1377 cm^−1^ correspond to the asymmetric and symmetric stretching vibrations of -COO^−^ coordinated to Fe^3+^, respectively; the peak at 1253 cm^−1^ is assigned to the C-N stretching vibration; and the peak at 765 cm^−1^ is due to the out-of-plane bending vibration of the aromatic C-H bonds. The composite UCNPs@NH_2_-MIL-53(Fe) fully retained all characteristic absorption bands of NH_2_-MIL-53(Fe): the N-H stretching vibration was observed at 3369 cm^−1^, the characteristic peaks of coordinated carboxylate were present at 1576 cm^−1^ and 1382 cm^−1^, and the C-N stretching vibration (1255 cm^−1^) and C-H bending vibration (769 cm^−1^) were clearly discernible. The slight blue shift of the N-H stretching vibration suggests the existence of weak interactions, such as hydrogen bonding, between the NH_2_-MIL-53(Fe) shell and the PVP-modified layer. The FTIR results unequivocally demonstrate that the NH_2_-MIL-53(Fe) shell was successfully grown onto the surface of PVP-modified UCNPs, confirming the successful construction of the UCNPs@NH_2_-MIL-53(Fe) core–shell structure.

Figure 9 presents the X-ray photoelectron spectroscopy (XPS) analysis, which further reveals the chemical states of each element. In the survey spectrum, the Na 1s peak at 1071.79 eV and the F 1s peak at 684.95 eV correspond to Na^+^ and F^−^ in the fluoride nanocrystal lattice, respectively. The high-resolution O 1s spectrum can be deconvoluted into two main components: the peak at 531.65 eV is assigned to the coordinated oxygen formed between Fe^3+^ and the carboxyl groups of the terephthalic acid ligand in the MOF framework; the peak at 533.48 eV primarily originates from physically adsorbed water molecules or hydroxyl groups on the material surface. Gd^3+^ possesses a half-filled 4f^7^ electron configuration, and its Gd 4d high-resolution spectrum exhibits complex multiplet splitting due to spin–orbit coupling and strong exchange coupling between the 4d photoelectron hole and the 4f electrons. Specifically, the three sub-peaks at 142.45, 144.47, and 148.90 eV belong to the Gd 4d_5_/_2_ multiplet, while those at 158.18, 158.99, and 161.03 eV belong to the Gd 4d_3_/_2_ multiplet. These split peaks all originate from Gd^3+^ in the NaGdF_4_ shell, confirming the successful construction of the intrinsic core–shell structure of the UCNPs from the XPS perspective. Meanwhile, the interfacial coordination interactions between the nanoparticles and NH_2_-MIL-53(Fe) subtly modulate the electron density distribution around Gd^3+^, as reflected by the changes in the relative intensities of these sub-peaks. In the N 1s spectrum, the dominant peak at 399.5 eV is assigned to the C-N bonds in the organic framework. The Y 3d spectrum displays a spin–orbit split doublet, with the Y 3d_5_/_2_ and Y 3d_3_/_2_ binding energies located at 159.03 eV and 161.06 eV, respectively, which can be unequivocally attributed to the characteristic signal of Y-F bonds in the NaYF_4_ lattice. The high-resolution Fe 2p spectrum exhibits typical multiplet splitting features of high-spin Fe^3+^. In the Fe 2p_3_/_2_ region, the spectrum can be fitted into two components at 709.72 eV and 712.83 eV, corresponding to different spin–orbit coupling final states; the corresponding components in the Fe 2p_1_/_2_ region appear at 722.60 eV and 728.26 eV. Additionally, the peaks at 718.52 eV and 733.18 eV are assigned to shake-up satellite peaks. The C 1s spectrum can be deconvoluted into three components: the peak at 284.78 eV is attributed to aromatic and alkyl carbon (C-C/C=C), that at 286.22 eV to C-N bonds, and that at 288.09 eV to carboxylate carbon (-COO^−^). Collectively, these XPS results fully support the successful construction of the core–shell structure and the chemical integrity of each component.

The XRD patterns of OA-UCNPs, PVP-UCNPs, and UCNPs@NH_2_-MIL-53(Fe) are shown in Figure 10A. The diffraction peaks of OA-UCNPs and PVP-UCNPs both matched well with the standard hexagonal-phase β-NaGdF_4_ (JCPDS No. 27-0699), exhibiting high crystallinity and no impurity phases, indicating that ligand exchange did not affect the crystal phase of the UCNPs. After the construction of the UCNPs@NH_2_-MIL-53(Fe) core–shell structure, the characteristic diffraction peaks of NaGdF_4_ were fully retained, demonstrating that the UCNP core maintained good phase stability during the growth of the MOF shell. No obvious diffraction peaks of NH_2_-MIL-53(Fe) were observed in the composite, which could be attributed to the relatively thin MOF shell with low crystallinity, as well as the strong diffraction signals of the UCNP core overshadowing the weak signals of the MOF.

As shown in Figure 10B, electron spin resonance (ESR) spectroscopy with 5,5-dimethyl-1-pyrroline N-oxide (DMPO) as the spin trap was employed to evaluate the capability of UCNPs@NH_2_-MIL-53(Fe) to generate hydroxyl radicals (•OH). At the initial time point (0 min), no discernible signal was observed. As the reaction proceeded (1, 5, and 10 min), a characteristic quartet signal with an intensity ratio of 1:2:2:1 gradually appeared, and its intensity increased monotonically, which can be unequivocally assigned to the DMPO-•OH spin adduct. This result directly confirms that the material can continuously catalyze the generation of •OH. As a highly cytotoxic reactive oxygen species, •OH can efficiently oxidize and damage lipids, proteins, and DNA within tumor cells, thereby inducing apoptosis, and acts as the core effector molecule in chemodynamic therapy (CDT). Collectively, the ESR findings intuitively validate that this nanoplatform possesses efficient •OH generation capability, demonstrating its significant application potential in tumor chemodynamic therapy.

The magnetization curves of NaYF_4_:Yb,Tm, NaYF_4_:Yb,Tm@NaGdF_4_:Eu, and UCNPs@NH_2_-MIL-53(Fe) were measured using a vibrating sample magnetometer (VSM) at room temperature over a magnetic field range of ±15 kOe to investigate their magnetic properties. As shown in Figure 10C, all three M-H curves passed through the origin without remanence or coercivity, indicating typical paramagnetic behavior for all samples. The magnetization of bare NaYF_4_:Yb,Tm was relatively weak; upon coating with the NaGdF_4_:Eu shell, the magnetization was significantly enhanced, which can be attributed to the intrinsic magnetic moments of abundant high-spin Gd^3+^ ions in the host matrix. After further growth of the NH_2_-MIL-53(Fe) shell, the magnetization underwent only a slight change. The above paramagnetic characteristics primarily originate from Gd^3+^ ions, endowing the material with potential as a T_1_-weighted magnetic resonance imaging (MRI) contrast agent. Combined with its NIR-responsive upconversion luminescence capability, this multifunctional nanoparticle is expected to realize dual-modal optical and magnetic resonance imaging, demonstrating promising application prospects in the field of multimodal biomedical diagnostic and therapeutic probes.

## 3. Materials and Methods

### 3.1. Materials

Lanthanide chloride hexahydrates, including YCl_3_·6H_2_O, YbCl_3_·6H_2_O, GdCl_3_·6H_2_O, EuCl_3_·6H_2_O, and TmCl_3_·6H_2_O (purity 99.99% each), 1-octadecene (ODE, >90%), oleic acid (OA, 99.7%), NaOH (97%), NH4F (98%), polyvinylpyrrolidone (PVP, average Mw ~2000), 2-aminoterephthalic acid (NH_2_-H_2_BDC, >90%), cyclohexane (≥99.0%), and methanol (99.5%) were all purchased from Aladdin Reagent Co., Ltd. (Shanghai, China). N,N-Dimethylformamide (DMF, biotechnology grade, 99% purity) and iron(III) chloride hexahydrate (FeCl_3_·6H_2_O, 99% purity) were purchased from Macklin Biochemical Co., Ltd. (Shanghai, China). Absolute ethanol (analytical grade) was obtained from Tianjin Kaitong Chemical Reagent Co., Ltd. (Tianjin, China). Commercially available purified water (Hangzhou Wahaha Group Co., Ltd., Hangzhou, China) was used throughout the experiments.

### 3.2. Nanomaterial Characterization

The morphology and particle size of the materials were analyzed using a Talos F200X G2 transmission electron microscope (TEM; Thermo Fisher Scientific, Waltham, MA, USA). The crystal structure was characterized with a SmartLab 9 X-ray diffractometer (XRD, Rigaku Corporation, Tokyo, Japan) at a scanning rate of 4°/min over a 2θ range of 5–90°. Fluorescence spectra were recorded on an F4600 Hitachi fluorescence spectrophotometer (Sains (Beijing, China) Instrument Co., Ltd.) equipped with a 40 W excitation source, scanning across 200–900 nm. The functional groups were identified using a Nicolet Summit Fourier-transform infrared spectrometer (FTIR; Thermo Fisher Scientific, Waltham, MA, USA; Class 1 laser product; 12 V DC, max 9 A). X-ray photoelectron spectroscopy (XPS) measurements were performed on a Thermo Scientific K-Alpha X-ray photoelectron spectrometer (Thermo Fisher Scientific, Waltham, MA, USA). All the above measurements were performed at room temperature. In addition, the particle size and size distribution were determined by analyzing TEM images with ImageJ software (version 1.54g; National Institutes of Health, Bethesda, MD, USA).

### 3.3. Preparation of NaYF_4_:Yb,Tm

YCl_3_·6H_2_O (0.2266 g), YbCl_3_·6H_2_O (0.0969 g), and TmCl_3_·6H_2_O (0.0012 g) were weighed in a molar ratio of Y^3+^:Yb^3+^:Tm^3+^ = 74.7:25:0.3 (total rare earth 1 mmol) and placed into a three-necked flask, followed by the addition of 6 mL of oleic acid (OA) and 15 mL of 1-octadecene (ODE). The mixture was heated to 150 °C and stirred under an inert atmosphere for 30 min to form a homogeneous clear solution and then cooled to 60 °C. Separately, 0.1 g of NaOH and 0.148 g of NH_4_F were dissolved in 10 mL of methanol, and the resulting solution was added dropwise into the flask. The temperature was raised to 110 °C and maintained for 20 min to remove methanol and water. Subsequently, a condenser was connected, and the mixture was further heated to 300 °C for 60 min under inert gas protection. After the reaction, the mixture was cooled to room temperature. The product was washed three times with a mixture of cyclohexane and absolute ethanol (1:5, *v*/*v*), collected by centrifugation, freeze-dried under vacuum, and stored at 4 °C.

### 3.4. Preparation of NaYF_4_:Yb,Tm@NaGdF_4_:Eu

GdCl_3_·6H_2_O (0.45 mmol), EuCl_3_·6H_2_O (0.05 mmol), OA (4 mL), and ODE (16 mL) were placed into a 100 mL three-necked round-bottom flask. The mixture was heated to 150 °C under continuous stirring in an argon atmosphere and maintained for 90 min to form a clear solution. After cooling to 80 °C, 0.5 mmol of NaYF_4_:Yb,Tm dissolved in 5 mL of cyclohexane was added. The temperature was then raised to 110 °C and held for 20 min to remove cyclohexane. Upon cooling to room temperature, NaOH (0.05 g) and NH_4_F (0.074 g) dissolved in 5 mL of methanol were introduced, and the mixture was stirred at 50 °C for 30 min. The solution was subsequently heated to 110 °C for 20 min to evaporate methanol and water and then further heated to 300 °C under an argon atmosphere and stirred for 60 min. After cooling to room temperature, the product was washed three times with a mixture of cyclohexane and absolute ethanol (1:5, *v*/*v*), collected by centrifugation, freeze-dried under vacuum, and stored at 4 °C.

### 3.5. Preparation of PVP-Coated NaYF_4_:Yb,Tm@NaGdF_4_:Eu

To create a suitable surface for the growth of MOFs, the OA ligands on the surface of the UCNPs were first removed, and the nanoparticles were then coated with polyvinylpyrrolidone (PVP). Specifically, 0.15 mmol of OA-capped NaYF_4_:Yb,Tm@NaGdF_4_:Eu was dispersed in 10 mL of absolute ethanol containing 1.2 mL of HCl (pH = 1) and sonicated for 30 min. After washing with absolute ethanol, the product was placed in 10 mL of acidic ethanol solution (pH = 4) and sonicated for 10 min. Following washing with deionized water, the nanoparticles were transferred to 30 mL of ethanol, and 0.03 g of PVP was added dropwise. The mixture was stirred magnetically at room temperature overnight (12 h). The product was collected by centrifugation, washed with absolute ethanol to remove excess PVP molecules, and lyophilized into a powder for further use.

### 3.6. Synthesis of NaYF_4_:Yb,Tm@NaGdF_4_:Eu@NH_2_-MIL-53(Fe)

To coat NH_2_-MIL-53(Fe) onto the NaYF_4_:Yb,Tm@NaGdF_4_:Eu nanoparticles, 20 mg of PVP-coated NaYF_4_:Yb,Tm@NaGdF_4_:Eu was dispersed in 30 mL of DMF, followed by the addition of 10 mg of FeCl_3_·6H_2_O. The mixture was stirred in the dark for 30 min to allow the adsorption of Fe^3+^ ions. Subsequently, 10 mg of 2-aminoterephthalic acid was added to the solution. The reaction mixture was heated to 65 °C and maintained at this temperature for 60 min. The product was collected by centrifugation, washed with ethanol and deionized water, and finally lyophilized into a powder for further use.

## 4. Conclusions

In this study, a three-layer core–shell nanocomposite, NaYF_4_:Yb,Tm@NaGdF_4_:Eu@NH_2_-MIL-53(Fe), was successfully constructed as a potential platform for NIR-light-mediated imaging and therapy, and its synthesis, morphological evolution, luminescence properties, and preliminary functional properties were systematically investigated. The hexagonal-phase NaYF_4_:Yb,Tm core was first synthesized and used as seeds for the epitaxial growth of the NaGdF_4_:Eu shell. The effects of key parameters—including the Eu^3+^ doping amount, reaction temperature, reaction time, and OA/ODE volume ratio—on the morphology and properties of the core–shell structure were examined to determine the optimal conditions for constructing the final composite. High-concentration Eu^3+^ doping or prolonged reaction time induced pronounced lattice strain, which drove anisotropic growth along the c-axis and led to a distinctive dumbbell-like morphology. This morphological evolution resulted from the combined effects of strain energy minimization and selective ligand passivation. The optimal conditions were determined to be 10 mol% Eu^3+^ doping, an OA/ODE volume ratio of 4:16, a reaction temperature of 300 °C, and a reaction time of 60 min. Under these conditions, the core–shell nanoparticles exhibited regular morphology, high crystallinity, and strong upconversion luminescence. Subsequently, PVP was modified onto the surface of the optimized core–shell nanocrystals, which served as a template to induce in situ uniform coating of the NH_2_-MIL-53(Fe) shell, ultimately yielding the three-layer core–shell structure. FTIR and XPS confirmed the successful surface chemical modification at each step. The as-prepared nanocomposite exhibited potential functional properties: the Gd^3+^ ions in the intermediate shell imparted paramagnetism to the material, suggesting potential as a T_1_-weighted MRI contrast agent; ESR spectroscopy indicated that the NH_2_-MIL-53(Fe) shell could catalyze the generation of hydroxyl radicals through the Fenton reaction, providing functional support for potential chemodynamic therapy.

## Figures and Tables

**Figure 1:**
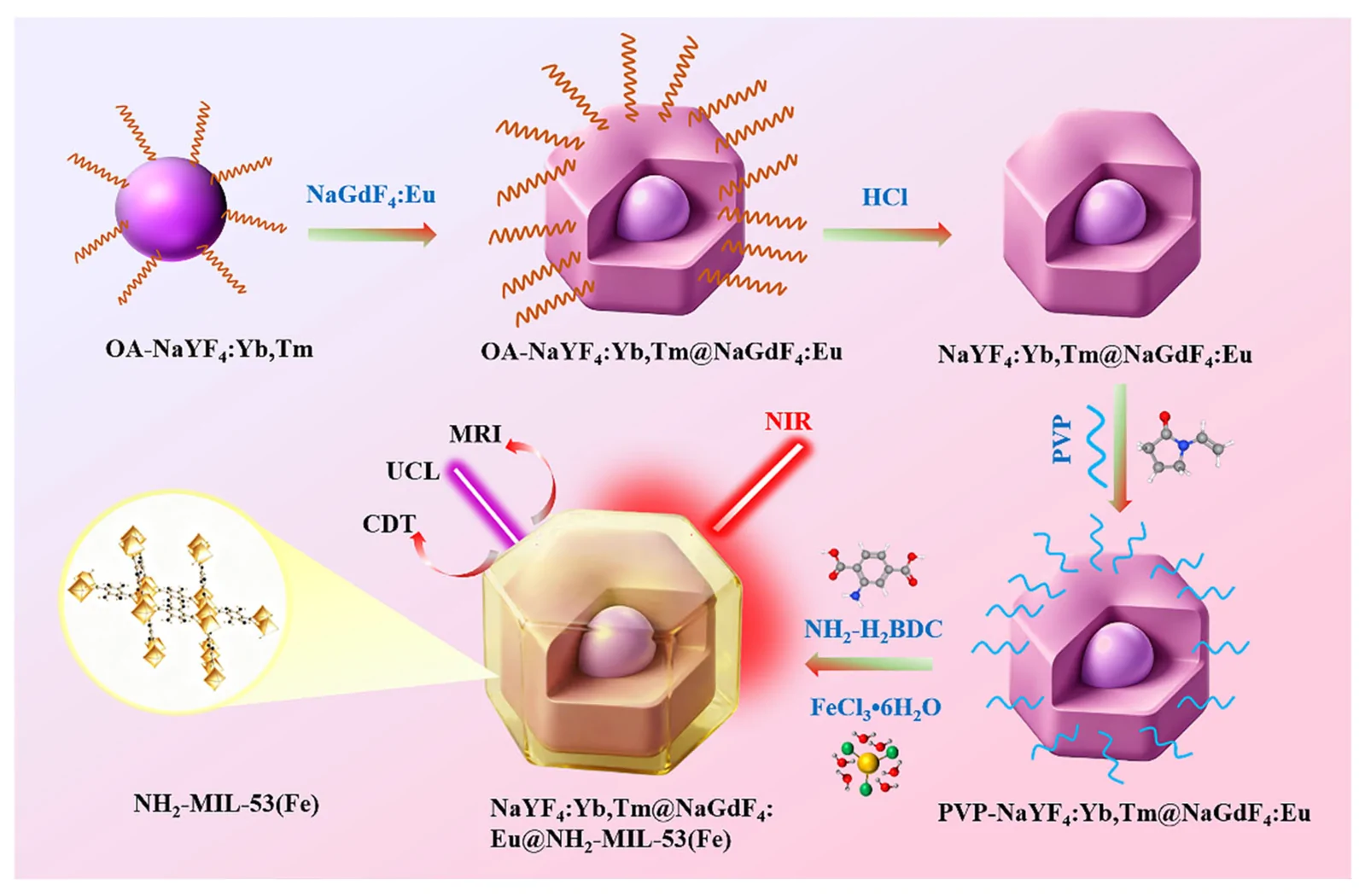
Schematic illustration of the formation process of NaYF_4_:Yb,Tm@NaGdF_4_:Eu@NH_2_-MIL-53(Fe) nanoparticles.

**Figure 2:**
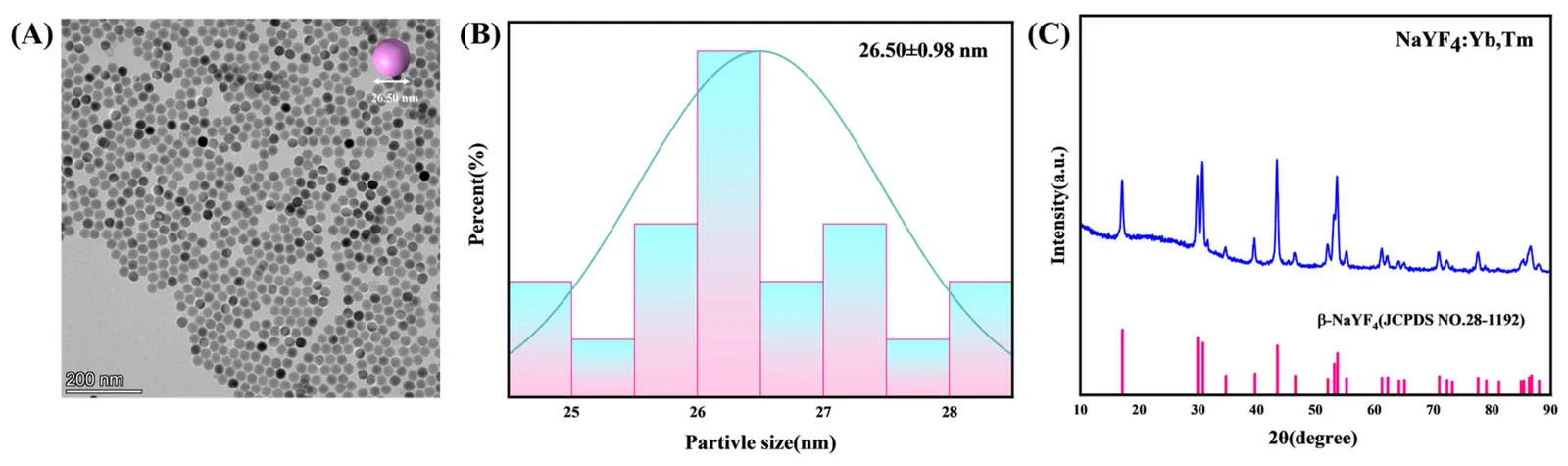
(**A**) TEM image of NaYF_4_:Yb,Tm; (**B**) particle size distribution of NaYF_4_:Yb,Tm; (**C**) XRD pattern of NaYF_4_:Yb,Tm.

**Figure 3:**
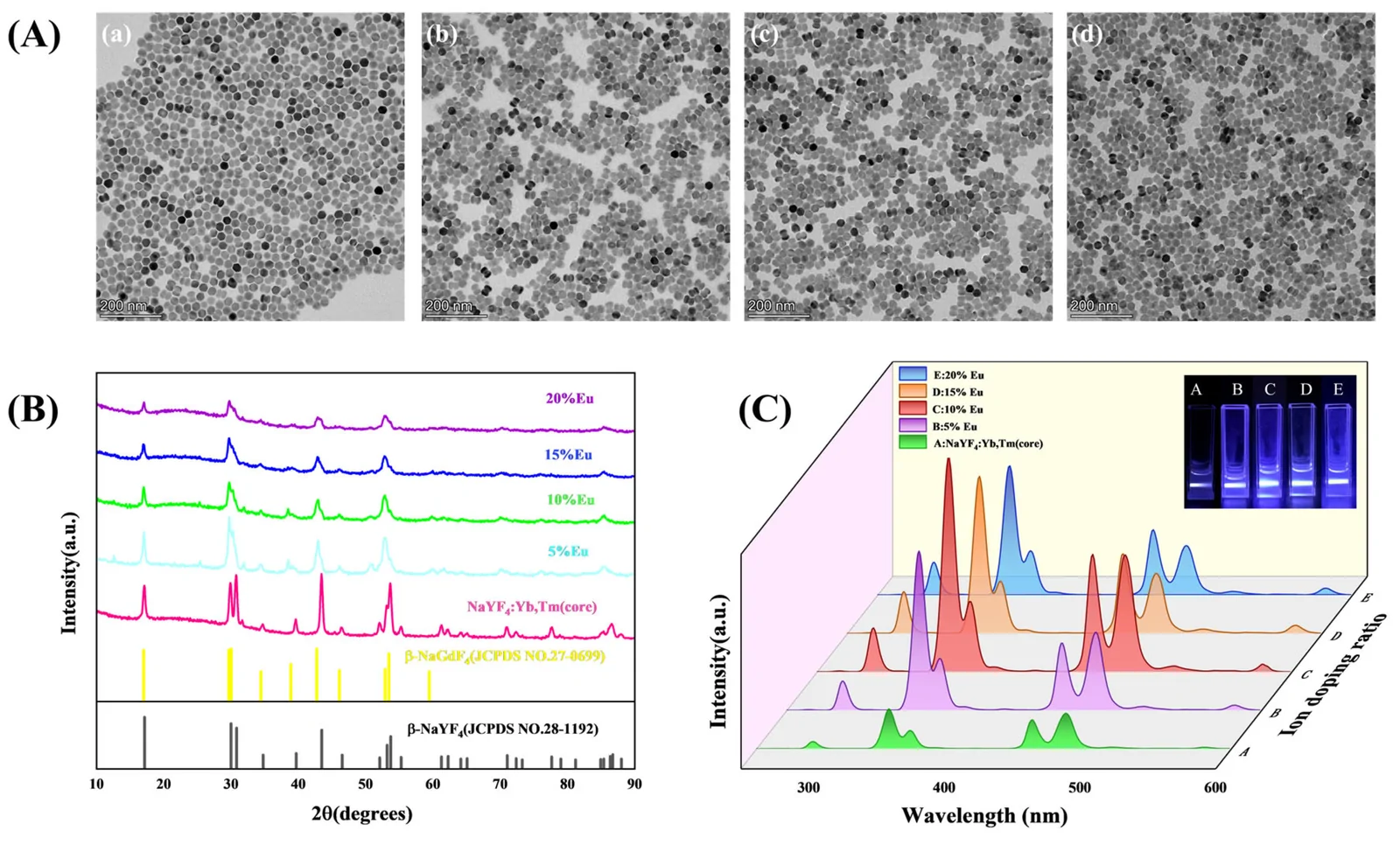
Characterization of NaYF_4_:Yb,Tm@NaGdF_4_:Eu synthesized with different Eu^3+^ doping amounts: (**A**) TEM images of the samples with Eu^3+^ doping amounts of (**a**) 5%, (**b**) 10%, (**c**) 15%, and (**d**) 20%; (**B**) XRD patterns; (**C**) upconversion fluorescence spectra under 980 nm excitation.

**Figure 4:**
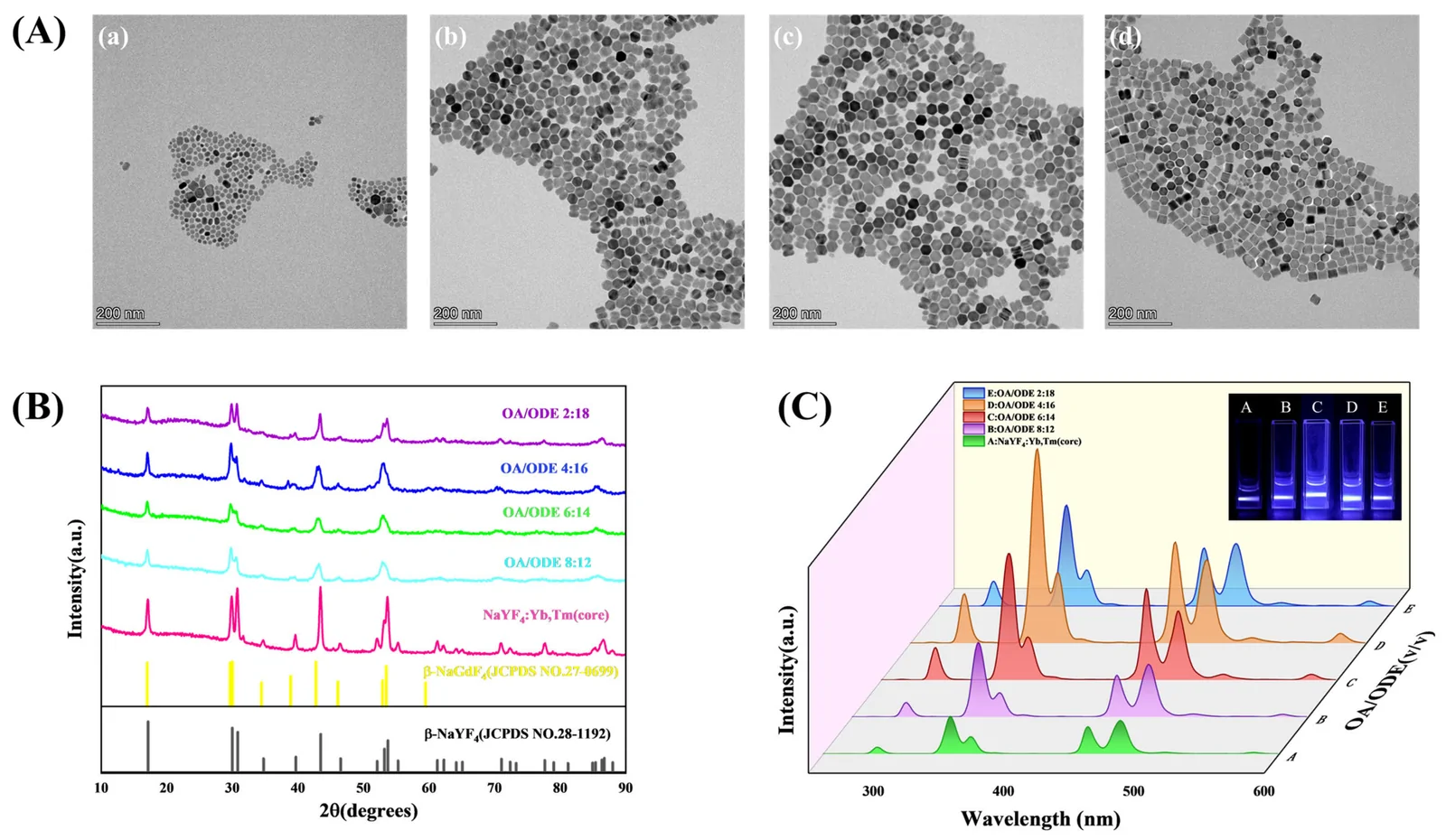
Characterization of NaYF_4_:Yb,Tm@NaGdF_4_:Eu synthesized at different OA/ODE volume ratios: (**A**) TEM images of NaYF_4_:Yb,Tm@NaGdF_4_:Eu synthesized at different OA/ODE volume ratios: (**a**) OA/ODE = 8:12, (**b**) OA/ODE = 6:14, (**c**) OA/ODE = 4:16, and (**d**) OA/ODE = 2:18; (**B**) XRD patterns; (**C**) upconversion fluorescence spectra under 980 nm excitation.

**Figure 5:**
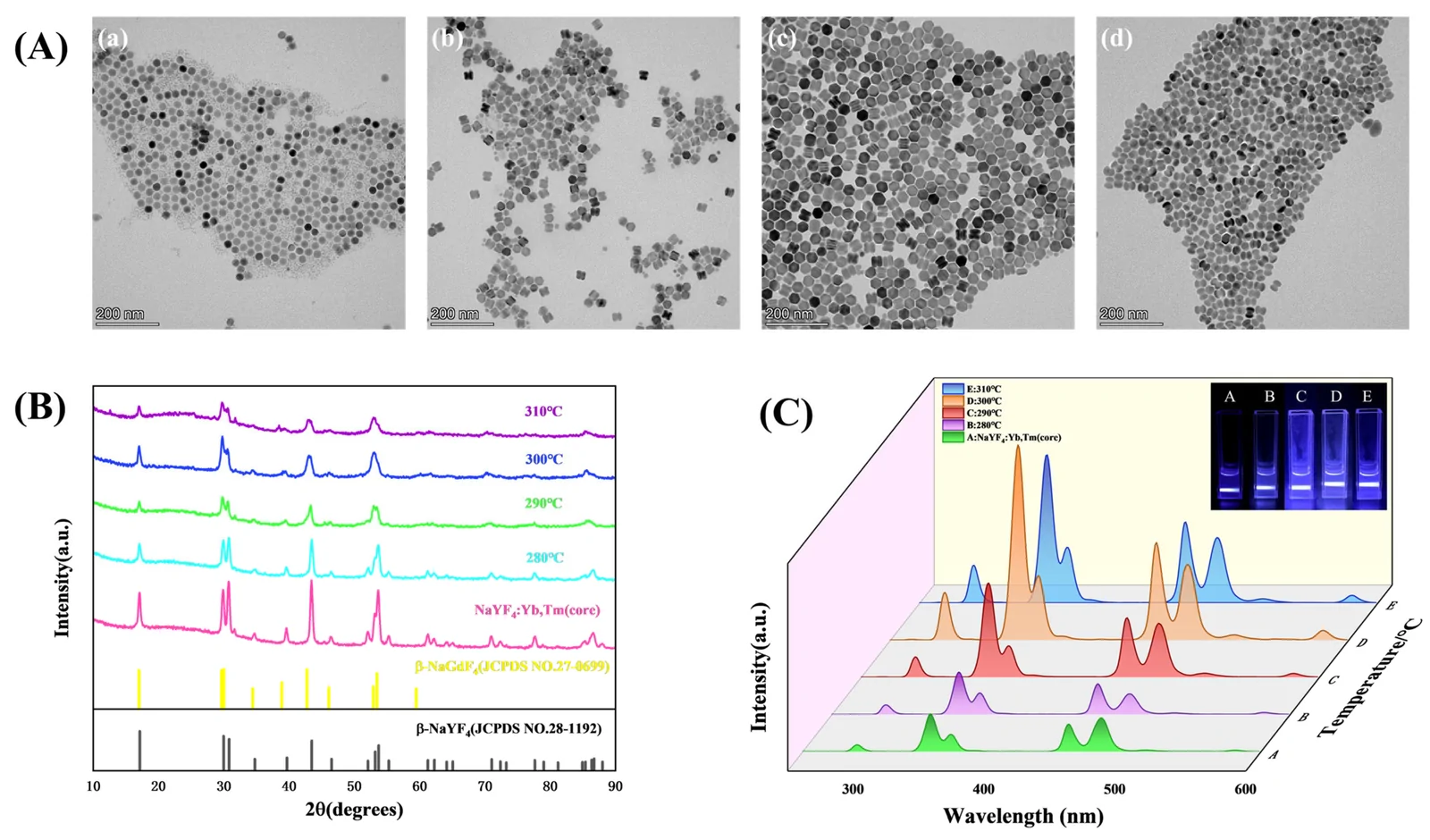
Characterization of NaYF_4_:Yb,Tm@NaGdF_4_:Eu synthesized at different reaction temperatures: (**A**) TEM images of the samples synthesized at (**a**) 280 °C, (**b**) 290 °C, (**c**) 300 °C, and (**d**) 310 °C; (**B**) XRD patterns; (**C**) upconversion fluorescence spectra under 980 nm excitation.

**Figure 6:**
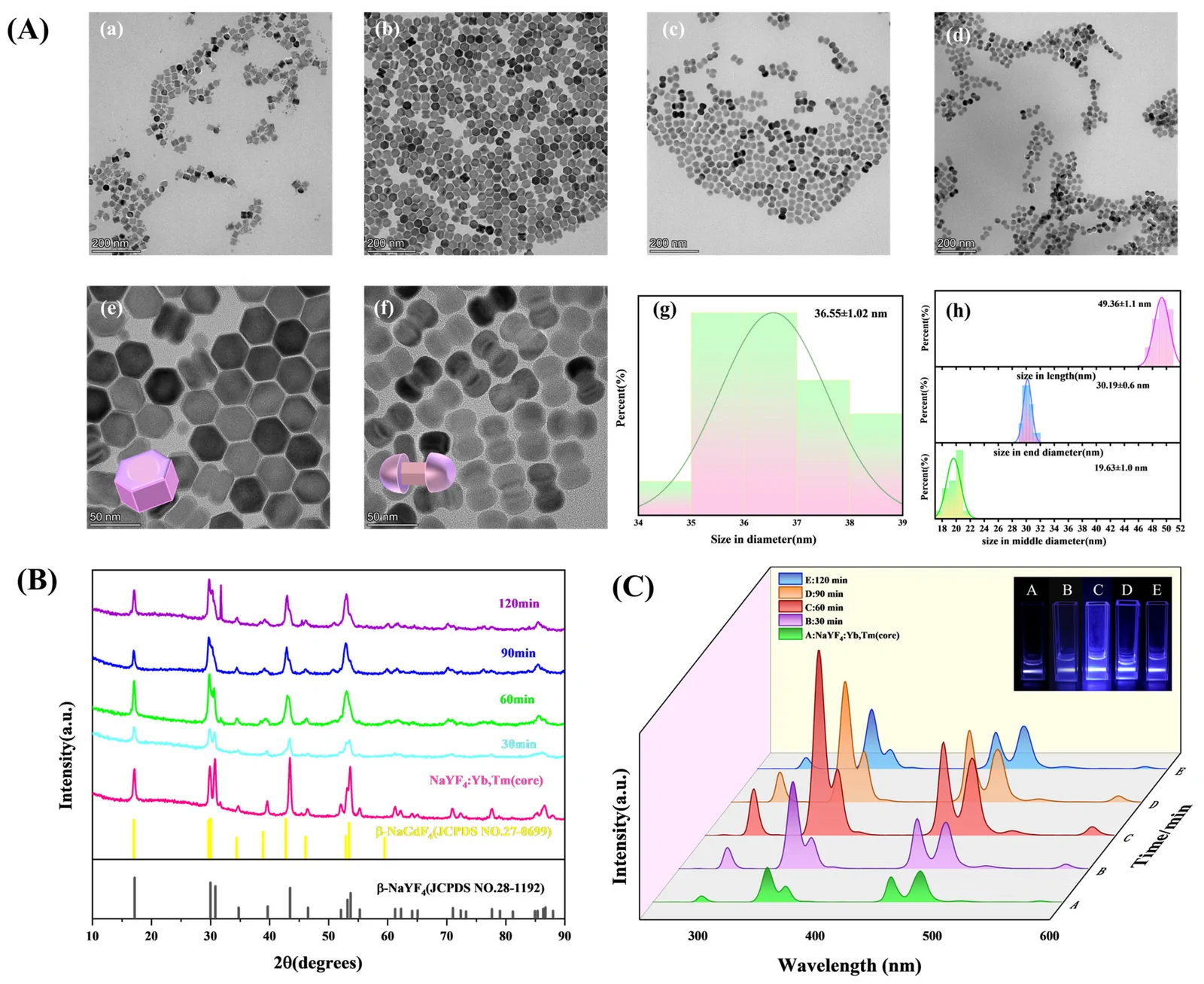
Characterization of NaYF_4_:Yb,Tm@NaGdF_4_:Eu synthesized at different reaction times: (**A**) TEM images of the samples obtained at different reaction times: (**a**) 30 min, (**b**) 60 min, (**c**) 90 min, and (**d**) 120 min; (**e**) high-resolution transmission electron microscopy (HRTEM) image and (**g**) diameter size distribution of the 60 min sample; (**f**) HRTEM image and (**h**) multi-parameter size distribution (length, end diameter, and middle diameter) of the 90 min sample. (**B**) XRD patterns of the products. (**C**) Upconversion fluorescence spectra of the samples under 980 nm excitation.

**Figure 7:**
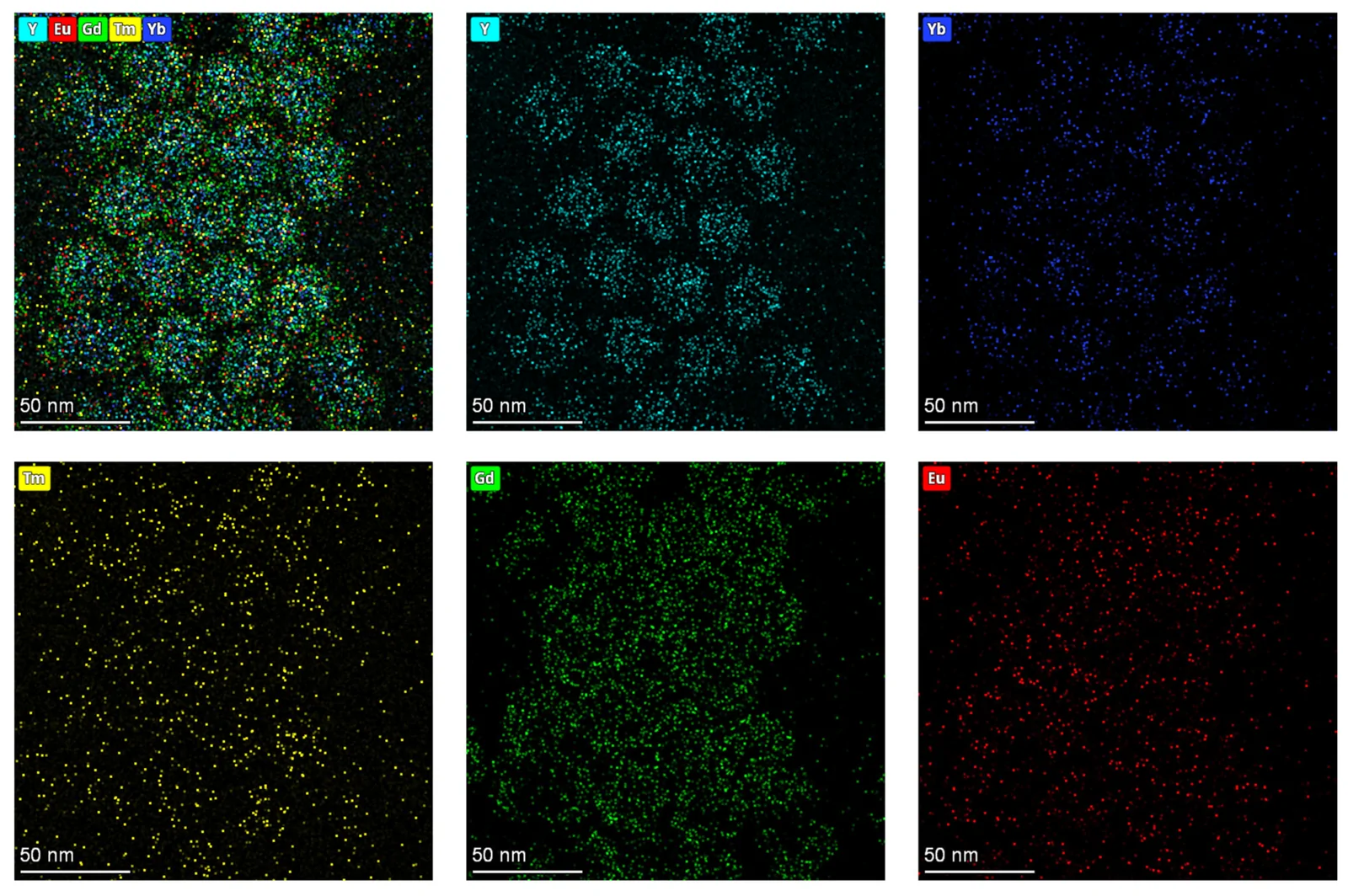
EDS elemental mapping of NaYF_4_:Yb,Tm@NaGdF_4_:Eu.

**Figure 8:**
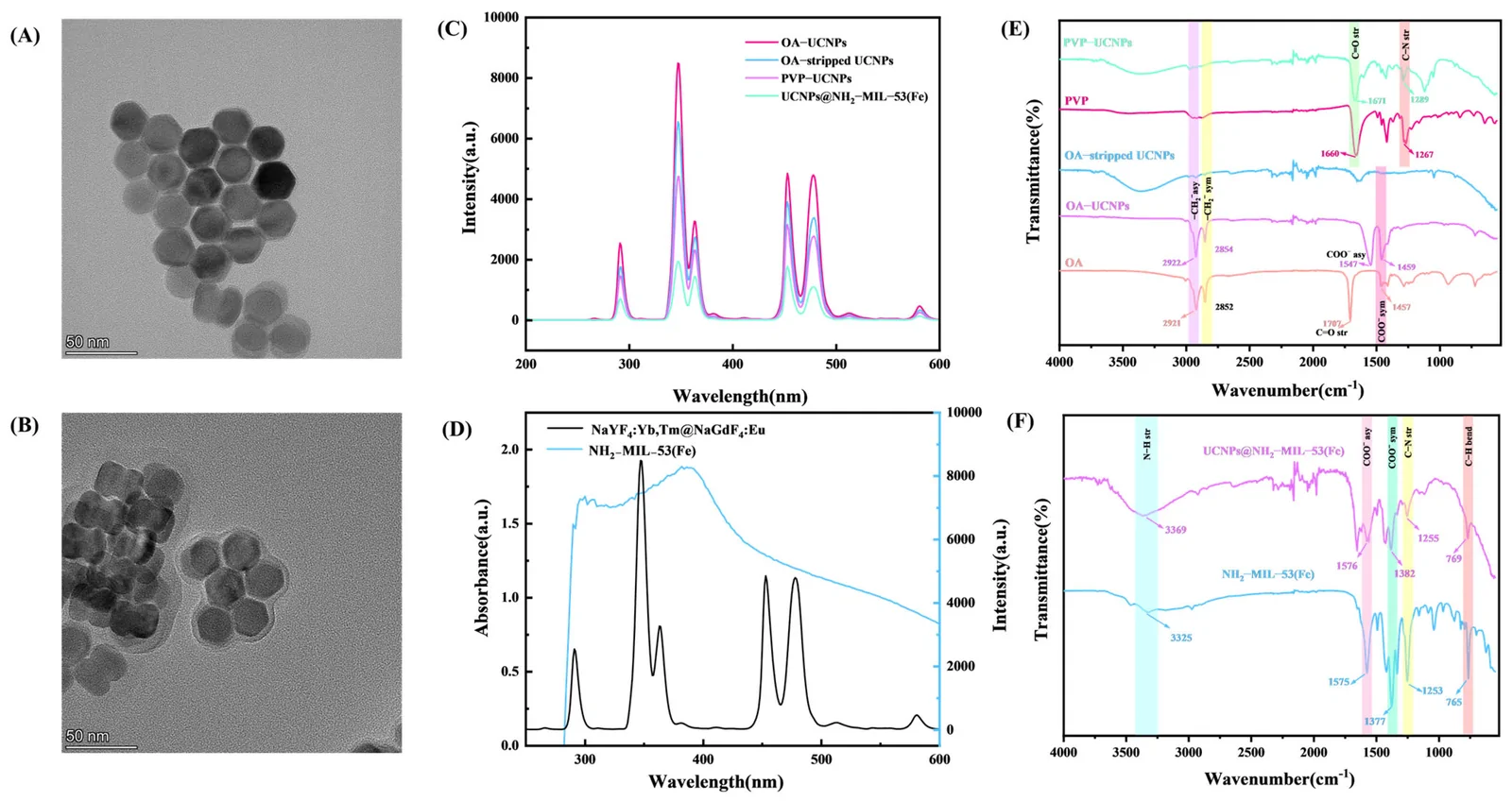
Comparative characterization of PVP-NaYF_4_:Yb,Tm@NaGdF_4_:Eu and UCNPs@NH_2_-MIL-53(Fe): (**A**) TEM image of PVP-NaYF_4_:Yb,Tm@NaGdF_4_:Eu; (**B**) TEM image of UCNPs@NH_2_-MIL-53(Fe); (**C**) upconversion fluorescence spectra of PVP-NaYF_4_:Yb,Tm@NaGdF_4_:Eu and UCNPs@NH_2_-MIL-53(Fe); (**D**) ultraviolet–visible (UV-vis) absorption spectrum of NH_2_-MIL-53(Fe) and upconversion emission spectrum of NaYF_4_:Yb,Tm@NaGdF_4_:Eu; (**E**) FTIR spectra of PVP-NaYF_4_:Yb,Tm@NaGdF_4_:Eu and UCNPs@NH_2_-MIL-53(Fe); (**F**) FTIR spectra of UCNPs@NH_2_-MIL-53(Fe) and NH_2_-MIL-53(Fe).

**Figure 9:**
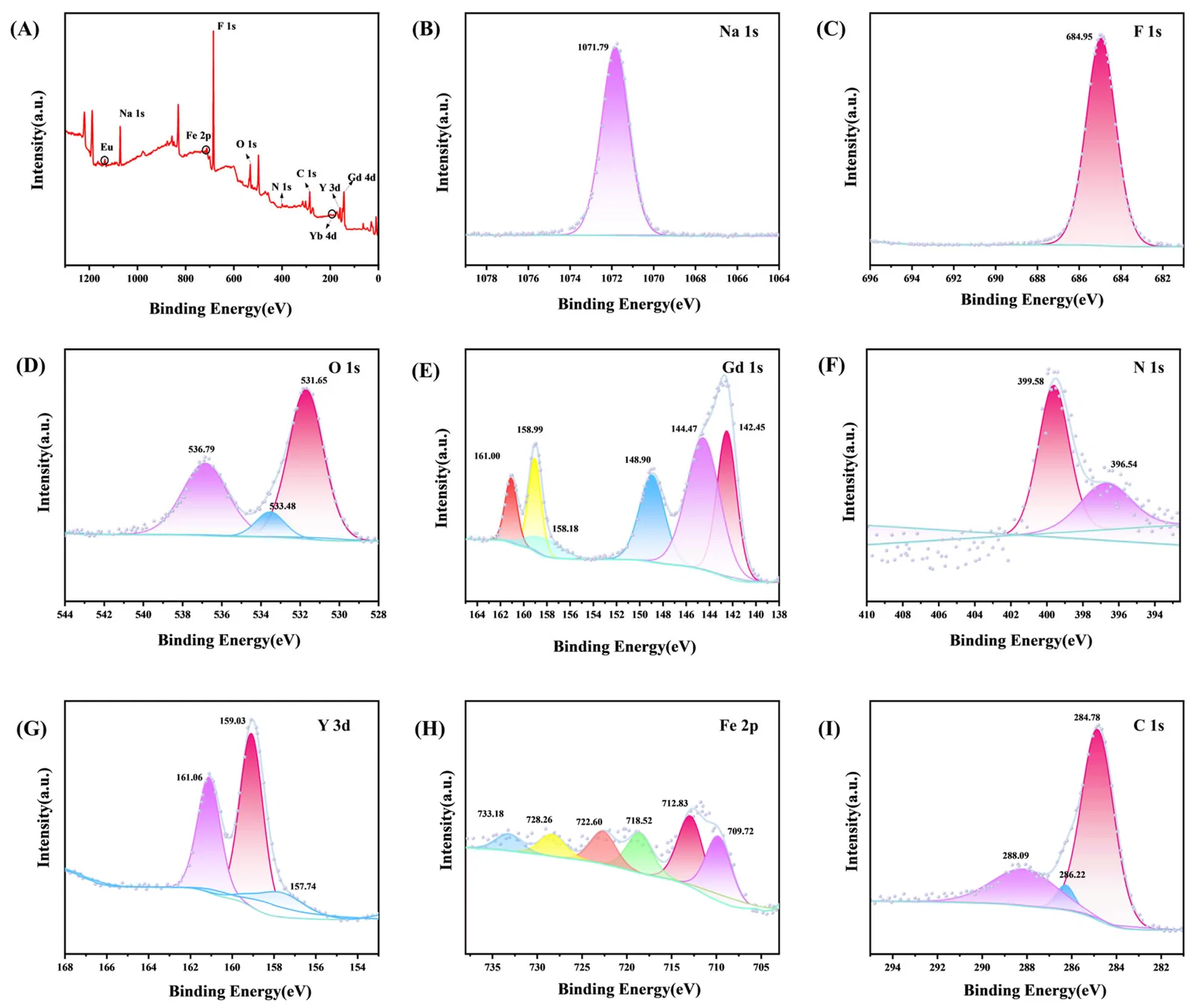
XPS spectra of NaYF_4_:Yb,Tm@NaGdF_4_:Eu@NH_2_-MIL-53(Fe): (**A**) survey spectrum; (**B**) Na 1s; (**C**) F 1s; (**D**) O 1s; (**E**) Gd 4d; (**F**) N 1s; (**G**) Y 3d; (**H**) Fe 2p; (**I**) C 1s.

**Figure 10:**
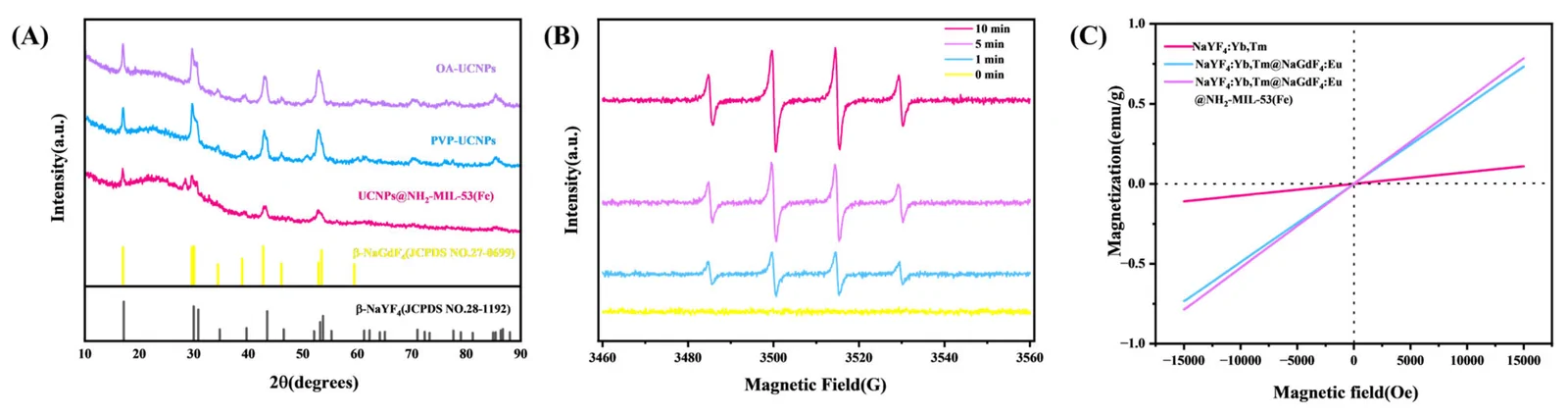
Phase, EPR, and magnetic property characterization of upconversion nanoparticles and their composite products: (**A**) X-ray diffraction (XRD) patterns of OA-UCNPs, PVP-UCNPs, and UCNPs@NH_2_-MIL-53(Fe), with the standard diffraction peaks of β-NaYF_4_ (JCPDS No. 28-1192) and β-NaGdF_4_ (JCPDS No. 27-0699) indicated at the bottom. (**B**) Electron paramagnetic resonance (EPR) spectra recorded at different reaction times (0 min, 1 min, 5 min, and 10 min). (**C**) Magnetization curves (VSM) of the NaYF_4_:Yb,Tm core, NaYF_4_:Yb,Tm@NaGdF_4_:Eu core–shell nanoparticles, and UCNPs@NH_2_-MIL-53(Fe) composite.

## Data Availability

The data presented in this study are available upon request from the corresponding author.
